# GABA and Glutamate Transporters in Brain

**DOI:** 10.3389/fendo.2013.00165

**Published:** 2013-11-11

**Authors:** Yun Zhou, Niels Christian Danbolt

**Affiliations:** ^1^The Neurotransporter Group, Department of Anatomy, Institute of Basic Medical Sciences, University of Oslo, Oslo, Norway

**Keywords:** GABA uptake, glutamate uptake, BGT1, GAT1, GAT3, GAT2, EAAT2, EAAT1

## Abstract

The mammalian genome contains four genes encoding GABA transporters (GAT1, slc6a1; GAT2, slc6a13; GAT3, slc6a11; BGT1, slc6a12) and five glutamate transporter genes (EAAT1, slc1a3; EAAT2, slc1a2; EAAT3, slc1a1; EAAT4, slc1a6; EAAT5, slc1a7). These transporters keep the extracellular levels of GABA and excitatory amino acids low and provide amino acids for metabolic purposes. The various transporters have different properties both with respect to their transport functions and with respect to their ability to act as ion channels. Further, they are differentially regulated. To understand the physiological roles of the individual transporter subtypes, it is necessary to obtain information on their distributions and expression levels. Quantitative data are important as the functional capacity is limited by the number of transporter molecules. The most important and most abundant transporters for removal of transmitter glutamate in the brain are EAAT2 (GLT-1) and EAAT1 (GLAST), while GAT1 and GAT3 are the major GABA transporters in the brain. EAAT3 (EAAC1) does not appear to play a role in signal transduction, but plays other roles. Due to their high uncoupled anion conductance, EAAT4 and EAAT5 seem to be acting more like inhibitory glutamate receptors than as glutamate transporters. GAT2 and BGT1 are primarily expressed in the liver and kidney, but are also found in the leptomeninges, while the levels in brain tissue proper are too low to have any impact on GABA removal, at least in normal young adult mice. The present review will provide summary of what is currently known and will also discuss some methodological pitfalls.

## Glutamate and GABA as Neurotransmitters

Gamma-aminobutyric acid (GABA) and glutamate are, respectively, the major inhibitory and the major excitatory neurotransmitters in the mammalian central nervous system (1–3), and are thereby involved directly or indirectly in most aspects of normal brain function including cognition, memory, and learning. They are exocytosed from nerve terminals, and it is currently debated whether they are also exocytosed from astrocytes [e.g., Ref. (4–6)]. When interpreting data in the literature, it is important to keep in mind that astrocytic preparations differ greatly depending on the source of the cells and the culturing conditions, and cultured astrocytes may differ substantially from mature brain astrocytes (5).

## The Importance of Cellular Uptake of GABA and Glutamate

Both GABA and glutamate exert their signaling roles by acting on receptors located on the surface of the cells expressing them [e.g., Ref. (7–13)]. Therefore it is the transmitter concentration in the surrounding extracellular fluid that determines the extent of receptor stimulation. It is of critical importance that the extracellular concentrations are kept low [e.g., Ref. (3, 14–16)]. This is required for a high signal to noise (background) ratio in synaptic as well as in extrasynaptic transmission.

Low extracellular levels can only be maintained by cellular uptake because there is no extracellular metabolism of GABA and glutamate [e.g., Ref. (17–24)].

For glutamate, there is another reason to keep the extracellular levels low. Excessive activation of glutamate receptors is harmful, and glutamate is thereby toxic in high concentrations [for review and references, see Ref. (3)].

## Early Characterization of GABA and Glutamate Uptake

It was soon realized that the mechanisms responsible for the uptake of GABA and glutamate are independent of each other (21, 25, 26) and that there is heterogeneity both within GABA uptake (27) and glutamate uptake (28–33). Uptake activity of both GABA and glutamate uptake was found to be present both in glial cells and in neurons [for review, see Ref. (1, 24, 34)]. The uptake processes are electrogenic and in the case of glutamate uptake it is driven by the ion gradients of K^+^ and Na^+^ [for review, see Ref. (35)] while the uptake of GABA is driven by the gradients of Na^+^ and Cl^−^ (35–39). The dependency of the transport process on the electrochemical gradients across the plasma membranes further implies that the uptake can reverse if the gradients are sufficiently weakened. In fact, during cerebral ischemia massive amounts of glutamate are released (40) and transporter reversal may be one of the mechanisms [e.g., Ref. (41–45)] albeit not the only one (3). Further, the transporters can operate as exchangers. The latter phenomenon complicated early attempts to study binding of glutamate to the uptake sites in membrane preparations (46–48), and it took some time before it was realized that transportable uptake inhibitors induce release of internal endogenous substrates by enabling heteroexchange [e.g., Ref. (3, 49)].

## Identification of GABA and Glutamate Transporters

The first neurotransmitter transporter to be molecularly identified was the GABA transporter (Table 1) now known as GAT1 (slc6a1). This was accomplished by purifying the protein in active form using reconstitution of transport activity as the assay to monitor the purification process (50). Based on peptide sequences derived from the pure protein, probes were synthesized and cDNA was successfully isolated from a rat brain library (51). This turned out to be the first member of a new family of transporters. Another three GABA transporters (GAT2, slc6a13; GAT3, slc6a11; BGT1, slc6a12) were subsequently identified (52, 53). The first cloning of BGT1 resulted from screening of a Madin–Darby canine kidney (MDCK) cell cDNA library for expression of a betaine transporter in *Xenopus* oocytes (54). BGT1 homologs were subsequently cloned from mouse brain (53), and from human brain (55) and kidney (56). In fact, the mammalian genome contains about 20 transporters with structural similarities to GAT1 (37, 38, 57). Interestingly, none of these were glutamate transporters.

**Table 1:** **Overview of the nomenclature of plasma membrane GABA transporters**.

Name adopted by HUGO (www.genenames.org)	Other names
GABA transporter 1 (GAT1; slc6a1)	Rat GAT1, human GAT1, mouse GAT1 (51, 52)
GABA transporter 2 (GAT2; slc6a13)	Rat GAT2, human GAT2, mouse GAT3 (52, 277)
GABA transporter 3 (GAT3; slc6a11)	Rat GAT3, hGAT-3, mGAT4, GAT-B (52, 277, 278)
Betaine-GABA transporter (BGT1; slc6a12)	Rat BGT1, rat GAT-4, rat NTBE, human GAT-4, mouse GAT2 (52–56, 180)
Taurine transporter (TAUT; slc6a6)	(279), (280)
Proton-coupled amino acid transporter 1 (PAT1; slc36a1)	Imino acid carrier, LYAAT-1, tramdorin 3 (281)

*The neurotransmitter transporter family (SLC6) comprises four GABA transporters as well as the taurine transporter (280). Both the taurine transporter and the proton-coupled amino acid transporter 1 (282) are able to transport GABA, but with low affinity (*K*_m_ > 1 mM) and are therefore usually not classified as “GABA transporters.” The nomenclature used here is the one adopted by the HUGO Gene Nomenclature Committee (283)*.

The first glutamate transporter (Table 2) to be isolated in active form (58) and localized (59, 60) was the one now known as EAAT2 [GLT-1; slc1a2; Ref. (61)]. Simultaneously, but independently of each other, three other research teams succeeded in cloning another two glutamate transporters using completely different approaches (62–64). The three human counterparts were quickly identified and named Excitatory Amino Acid Transporter (EAAT) 1–3 (65). Another two glutamate transporters were found later: EAAT4 (66) and EAAT5 (67). All the EAATs catalyze Na^+^- and K^+^-coupled transport of l-glutamate as well as l- and d-aspartate, but not d-glutamate. Further, down-regulation of glial glutamate transporters after glutamatergic denervation suggested complex regulation (68). Glutamate transporter expression turned out to be regulated via several different pathways and neurons were found to influence astroglial expression levels [e.g., Ref. (69–72)]. In fact, there is regulation on apparently all levels from transcription to posttranslational modification and trafficking (73, 74). This degree of complexity suggested that the transporters might have more roles than simply representing drainage and re-cycling systems [for review, see Ref. (3, 73, 75–79)]. Pharmacological manipulation of transporter expression or function would be highly interesting from a therapeutic point of view (80). A spider toxin has been found to enhance EAAT2 transport activity (81), but the compound responsible has not yet been identified. Recently, it was discovered EAAT2 expression can be increased by β-lactam antibiotics (82, 83), and that finding has got considerable attention.

**Table 2:** **Overview of the nomenclature of plasma membrane glutamate transporters**.

HUGO name	Other names
Excitatory amino acid transporter 1 (EAAT1; slc1a3)	GLAST (63–65)
Excitatory amino acid transporter 2 (EAAT2; slc1a2)	GLT-1 (61, 65)
Excitatory amino acid transporter 3 (EAAT3; slc1a1)	EAAC1 (62, 65, 211)
Excitatory amino acid transporter 4 (EAAT4; slc1a6)	(66)
Excitatory amino acid transporter 5 (EAAT5; slc1a7)	(67)

*Glutamate transporters do not belong to the slc6-family, but to the slc1-family (high-affinity glutamate and neutral amino acid transporter family). Although there are several proteins with ability to transport glutamate, the term “glutamate transporter” is usually used to describe the five “High-Affinity Glutamate Transporters” also called “Excitatory Amino Acid Transporters (EAATs).” The actual meanings of the acronyms (GLAST, glutamate–aspartate transporter; GLT-1, glutamate transporter; EAAC, excitatory amino acid carrier; EAAT, excitatory amino acid transporter) are not important, as they do not reflect functional differences among the transporters. The nomenclature used here is the one adopted by the HUGO Gene Nomenclature Committee (283)*.

## Functional Properties of GABA Transporters

The molecular functioning of GAT1 has been extensively studied (84–92), but there are also data on the other three GABA transporters (93–100). GAT1 and GAT3 are coupled to both sodium and chloride. Like for the glutamate transporters (see below), there is also uncoupled transport (101–103). The affinities for GABA vary greatly. The reported *K*_m_ values for the mouse isoforms are 0.8, 8, 18, and 80 μM, respectively, for GAT3, GAT1, GAT2, and BGT1 (52, 53, 104). Nipecotic acid and β-guanidinopropionate inhibit the GAT1–3, but not the taurine transporter (105). GAT2 (slc6a13) transports β-alanine and also taurine with *K*_m_ of 28 and 540 μM, respectively (52, 106). Mouse BGT1 (slc6a12) transports betaine with a *K*_m_ of about 200 μM, but no significant transport of β-alanine could be detected (53). Mouse GAT2 and GAT3 are more potently inhibited by isoserine, β-alanine, and hypotaurine than GAT1 and BGT1 (107). Tiagabine is highly selective for GAT1 (24, 108, 109). Recently, new functional assays have been developed for compound screening (110, 111) leading to development of several new compounds (112–116).

## Functional Properties of Glutamate Transporters

Most of the reported *K*_m_ values of EAAT2 for glutamate are at around 20 μM and the affinities of EAAT1 and EAAT3 differ from EAAT2 with a factor of <2 (3), while the affinities of EAAT4 and EAAT5 are, respectively, one order of magnitude higher and lower (3, 65–67). Stoichiometry of the transport mediated by EAAT1–3 is exchange of one internal potassium ion with the following external substrates: one glutamate, three sodium ions, and one hydrogen ion (117–119). The coupling to three sodium ions makes these transporters less prone to reversal than the GABA transporters which are coupled to two sodium ions. In addition to the coupled (stoichiometric) transport, there are uncoupled fluxes. Thus, the transporters also function as chloride channels (66, 117, 120–122). EAAT4 and EAAT5 have the largest chloride conductance, and may function more as inhibitory glutamate receptors than as transporters (123, 124). In addition, a general feature of sodium coupled transport appears to be transport of water (125, 126). Obviously, these transporters are complex molecules, and it is important to determine their exact structure. Although the mammalian transporters have not yet been crystallized, a detailed picture is emerging (127, 128). The mammalian EAAT2 and EAAT3 proteins are believed to be homotrimers where the subunits are non-covalently connected (129). This is supported by recent evidence including crystallization of a bacterial glutamate transporter (130, 131). However, crosslinking studies indicate that there may be differences between the EAAT subtypes (123). These proteins are integral membrane proteins and they depend on the lipid environment. For instance, the GABA transporters, at least GAT1, need cholesterol to be fully active (132). EAAT2 is more robust, but is influenced by fatty acids such as arachidonic acid (133–135) and oxidation (136, 137). Arachidonic acid elicits a substrate-gated proton current associated with the glutamate transporter EAAT4 (138, 139).

All the five EAATs transport l-glutamate and dl-aspartate with high affinities (3, 140). There are some important differences, however. One of them is that EAAT3 transports cysteine (141). Another is that EAAT2 is blocked by kainate and dihydrokainate (65). Importantly, kainate analogs block both net uptake and exchange [for the importance of this, see Figure 5 in Ref. (3)] while most other inhibitors are substrates. Recently, a pan-EAAT blocker was developed by Shimamoto and co-workers. They synthesized a series of compounds (TBOA and variants) based on aspartate (142). The only known biological effect of these compounds is to bind to EAATs with higher affinity than glutamate (143, 144) and they do not interact with ASCT2 (145). An EAAT1 selective inhibitor has also been developed (146).

## Localization and Function – Numbers Matter

Still, most of the data on protein distribution relies on immunohistochemistry. Unfortunately, validation of the specificity of immunochemical labeling is difficult and the literature reflects that [for detailed discussion, see Ref. (147–149)]. The most difficult part is to obtain good negative controls. When studying human samples, post-mortem proteolysis may complicate interpretation because the termini of EAAT1 and EAAT2 are rapidly proteolyzed (150–152). Post-mortem changes affect GAT3 more than GAT1 (153). It is a good idea to use additional methods, including *in situ* hybridization and Western blotting. For instance, Western blotting can be used to validate regional and temporal differences in labeling intensity. However, there are pitfalls here too. One of them is that non-transporter proteins may interfere with the binding of transporters to the blotting membranes causing underestimation of expression levels (106, 154).

The presence of a protein is one thing. But to be physiologically relevant, sufficient numbers of molecules must be present. The number of molecules needed to accomplish a given task depends on what that task is. This consideration is particularly relevant for neurotransmitter transporters as the transport process is fairly slow. The cycling time of EAAT2 and EAAT3 are about 30 glutamate molecules per second at *V*_max_ (14, 155, 156). EAAT5 is even slower and is reported to behave as a slow-gated anion channel with little glutamate transport activity being more than an order of magnitude slower than EAAT2 (157). The cycling time for the GABA transporters has not been determined, but is believed to be similar to that of EAAT2.

The TBOA glutamate uptake blocker (143, 158) showed that there is a rapid extracellular turnover of glutamate (159). Despite that, the resting levels of extracellular glutamate in normal brains are low, possibly as low as 25 nM (160) which is 0.1–0.2% of the reported *K*_m_ values for glutamate uptake (see above). At this concentration <0.1% of the glutamate transporter molecules are expected to be actively transporting. Consequently, if ambient concentrations of 25 nM shall be maintained, then there must be so many EAAT molecules that 0.1% of them is sufficient to keep up with the release. In fact, this is what has been determined experimentally (123, 155, 161, 162). Buffering synaptically released glutamate on a submillisecond time scale is just as demanding (163).

Also the ambient GABA levels around synapses are low; probably well below 1 μM (164–166) and thereby below the *K*_m_ of GAT3. The low levels mean that GABA is removed efficiently and down to a level where BGT1 (and GAT2) is much less efficient that GAT1 and GAT3. This illustrates the point that it is not interesting whether a few BGT1 molecules can be found or not, but whether there are enough of them to make a difference. Because BGT1 has lower affinity (see above) and is expressed in the brain at much lower levels than that GAT1, it cannot contribute to GABA inactivation and does not affect seizure thresholds (167).

## Distribution of GABA Transporters

**Figure 1:**
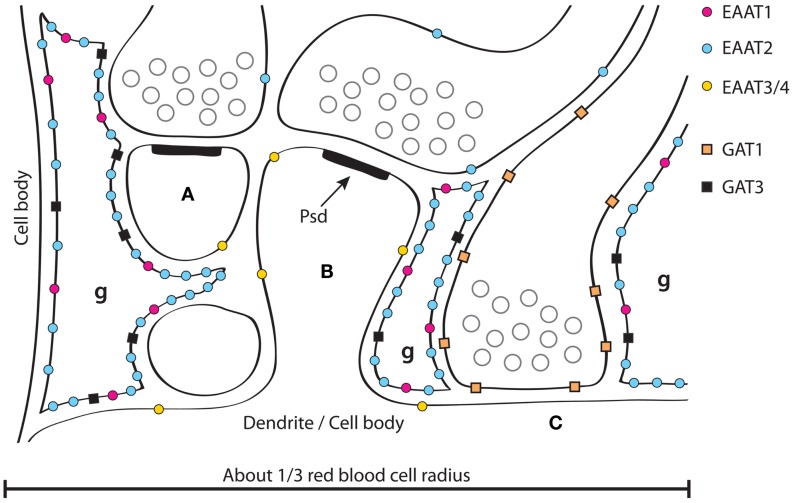
**A schematic illustration of GABA and glutamate transporter distributions around synapses in the hippocampus**. Two glutamatergic synapses **(A,B)** are shown forming synapses asymmetric specializations with prominent post synaptic densities (PSD, one of which is labeled). GABAergic synapses **(C)** are often onto dendritic trunks rather than spines, and the synaptic specializations are typically symmetric. Three fine astrocyte branches are indicated (g). Note that the synapses in the hippocampus are usually not surrounded by astrocytes, but rather contacted by an astrocyte (like a finger pointing to it, and typically from the postsynaptic side). Also note that there are no astrocytes between synapse **(A,B)**. About 1/3 of neighboring synapses in the hippocampus have no astrocytes between them in contrast to the molecular layer of the cerebellum where most synapses onto spines are typically completely surrounded by astrocytes (Figure 2) and thereby isolated from their neighbors (162, 275). Glutamate and GABA transporters are indicated. EAAT1 (184, 185) and GAT3 (153, 172, 175–177) are selective for astrocytes, while EAAT2 is predominantly expressed in astrocytes (59), but there is also some (about 10%) in hippocampal nerve terminals (229). This has some resemblance to GAT1 as GAT1 is mostly neuronal (170–173), but with some expression in astrocytes; particularly in the thalamus (172). There is more EAAT2 than EAAT1 in the hippocampus and the other way around in the cerebellum (162, 184). EAAT3 is selective for neurons, but is expressed at levels two orders of magnitude lower than EAAT2 and is targeted to dendrites and cell bodies (193) (Copyright: Neurotransporter.org; Reproduced with permission).

**Figure 2:**
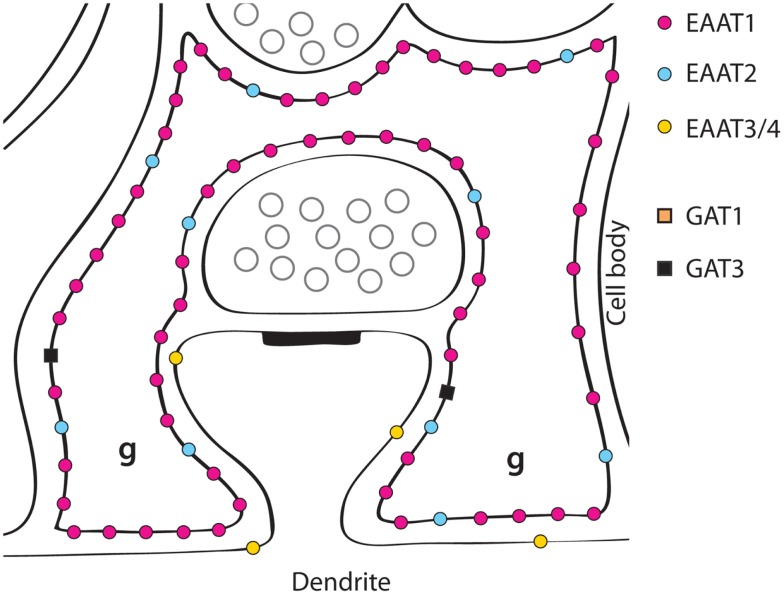
**A schematic illustration of glutamate transporter distributions around parallel fiber synapses on Purkinje cell spines in the molecular layer of the cerebellum**. EAAT1 is expressed in the astrocytes (Bergmann glial cells) at very high levels, and about six times higher than EAAT2 (162, 184). Most of the EAAT4 protein is found in cerebellar Purkinje cells (the glia-covered parts of the membranes of Purkinje cell dendrites), but low levels are also found in scattered neurons in the neocortex (123). EAAT3 is also found in the Purkinje cell dendrites, as well as in the other neuron types present, but at low levels (193) (Copyright: Neurotransporter.org; Reproduced with permission).

**Figure 3:**
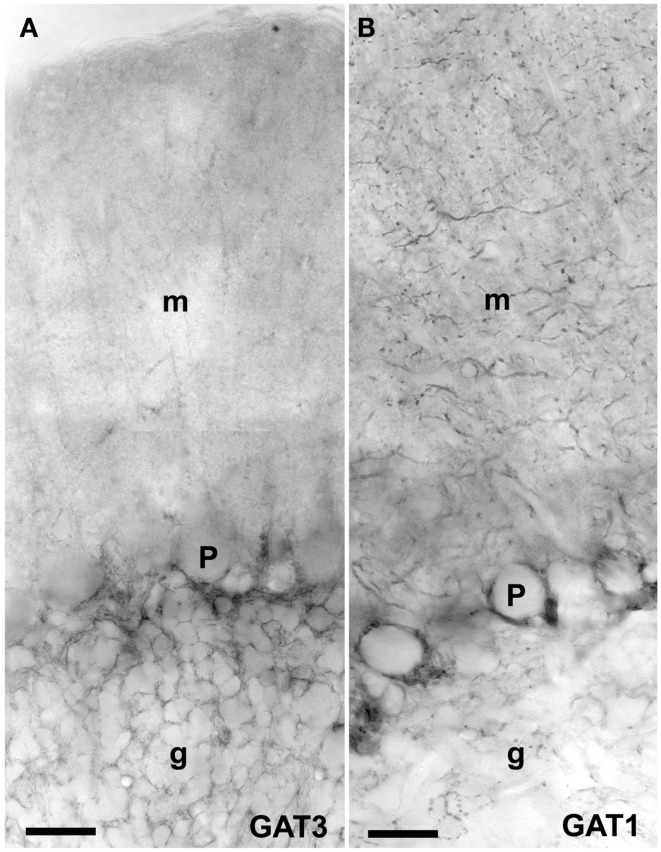
**Peroxidase labeling of GAT3 (A) and GAT1 (B) in the cerebellum [as described Ref. (175, 276)]**. GAT1 (slc6a1) and GAT3 (slc6a11) are the two GABA transporters that are functionally relevant for brain function, and these transporters are not expressed in the liver and kidney (181). GAT1 is mostly localized in axonal terminals in molecular layer (m) and GAT3 is in astroglial processes in granular layer (g). Note very high levels of GAT1 in basket cell terminals around the Purkinje cells (P). Scale bar: 20 μm (Copyright: Neurotransporter.org; Reproduced with permission).

The purified rat GAT1 protein (50) was used to generate the first antibodies to a GABA transporter and these were used to localize GAT1 in young adult rat brain tissue (168). These antibodies were probably selective for GAT1, but reactivity toward the other GATs were never tested. Nevertheless this antibody did not label cell bodies and the strongest labeling was found in GABAergic terminals. Basket cell terminals around the base of the Purkinje cells, for instance, were strongly labeled. Most GABAergic terminals were labeled, but with two notable exceptions: striatonigral and Purkinje cell axon terminals. There was also some labeling of astrocytes. These findings were confirmed by antibodies produced to synthetic peptides (169, 170) and thereby to sequences known to differ between GABA transporter subtypes (170–173). In thalamus, however, GAT1 was not found in terminals. All the immunolabeling was in astrocytes together with GAT3 (172). GAT1 is present in neuronal cell bodies for a short time during development (174). In contrast to GAT1, GAT3 (Figures 1–3) is considered to be selectively expressed in astrocytes throughout the CNS [e.g., Ref. (153, 172, 175–177)]. The highest GAT1 levels are in the cerebral cortex while the highest GAT3 levels are in the brainstem (178, 179).

**Figure 4:**
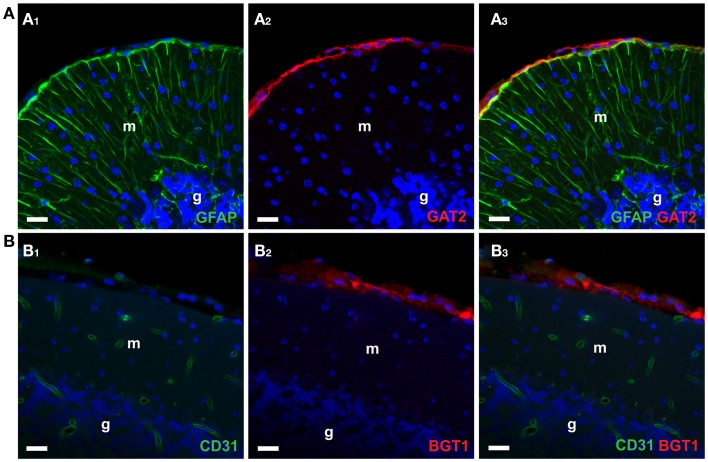
**Confocal imaging of GAT2 and BGT1 protein in the cerebellum [as described Ref. (106, 181)]**. (**A1–A3)** Show double labeling for GAT2 (red) and the astroglial marker GFAP (green). (**B1–B3)** Show double labeling for BGT1 (red) and the endothelial marker CD31 (green). The nuclei were visualized in blue by DAPI. Note that both GAT2 and BGT1 are found at the leptomeninges external to glia limitans. Also note that there is no co-localization of GAT2 with GFAP, and no co-localization of BGT1 with endothelial marker. In fact, BGT1 (106, 167) and GAT2 (181) are hardly expressed in the brain at all, but are highly expressed in the liver and kidney. Abbreviations: m, molecular layer; g, granular layer. Scale bar: 20 μm (Copyright: Neurotransporter.org; Reproduced with permission).

In contrast, GAT2 and BGT1 are predominantly expressed in hepatocytes in the liver and kidney (52, 53, 106, 180, 181). Within the skull (Figure 4A), GAT2 is found only in the leptomeninges and some large blood vessels (169, 181, 182) while BGT1 (Figure 4B) is expressed in the leptomeninges (106, 178).

## Distribution of Glutamate Transporters

When the first polyclonal and monoclonal antibodies were raised against the purified EAAT2 protein (58), it was immediately clear that EAAT2 is highly expressed in astrocytes in all parts of the brain and spinal cord. The highest levels were found in the hippocampus and the neocortex (59, 60). Soon thereafter antibodies were raised to synthetic peptides representing parts of the various subtypes. This made it easier to ensure that the antibodies were subtype specific. The conclusions on the distribution of EAAT2 were confirmed (183, 184), while EAAT1 was localized for the first time (184) and also this protein was found in astrocytes throughout the central nervous system (184–188). With immunogold and electron microscopy, it was shown that both EAAT1 and EAAT2 are preferentially targeted to the plasma membranes, and that plasma membranes facing neuropil have higher densities than those facing cell bodies, other astrocytes, and pia mater (189). Quantitative immunoblotting of brain tissue extracts compared with known amounts of purified glutamate transporters showed that EAAT2 protein represents about 1% of the total forebrain protein and that it is about four times more abundant than EAAT1 in the hippocampus and six times less abundant than EAAT1 in the cerebellum (162). The high expression levels are part of the reason why the first post-embedding immunogold electron micrographs of EAAT1 and EAAT2 (189) as well as EAAT4 (123) were so clear. Of course, good antibodies and good tissue preparation are key factors, but to get good immunogold images, there must also be a large number of molecules in the plane of the section.

Immunoadsorption of transport activity revealed that EAAT2 represent about 95% of the total glutamate uptake activity in young adult forebrain tissue (59, 129). Deletion of the EAAT2 gene in mice confirmed this conclusion as the glutamate uptake activity was reduced to 5% compared to wildtype mice (190–193) without changing the expression of other glutamate transporters, glutamine synthetase (GS), and glutamate GluR1 receptors (194). Further, electrophysiological recordings of glutamate transporter currents from hippocampal astrocytes and from human embryonic kidney 293 cells expressing human EAAT2 are statistically indistinguishable suggesting that the transporter currents in astrocytes result from EAAT2 or a functionally identical isoform (155).

EAAT1 (Figures 1 and 2) is the predominant glutamate transporter in the cerebellum (162, 195), the inner ear (196, 197), the circumventricular organs (198), and in the retina [Ref. (199–204); for review, see Ref. (205)].

EAAT2 and EAAT1 are the only glutamate transporters expressed in brain astrocytes as both EAAT3 (193, 206) and EAAT4 (123, 207, 208) are neuronal. Within the CNS, EAAT5 is preferentially expressed in the retina and expression in the brain is very low. It is interesting to note that also in insects (at least in the cabbage looper *Trichoplusia ni*) glial cells have high densities of glutamate transporters in their plasma membranes (209, 210).

EAAT3 is a neuronal transporter as originally suggested (62, 183, 211) and is not expressed in glial cells (193, 206). It appears to be expressed in the majority if not all neurons throughout the CNS, but is selectively targeted to somata and dendrites avoiding axon terminals (193, 206). Within the CNS, it is found in the highest concentration in the hippocampus, but the total tissue content in young adult rat brains is about 100 times lower than that of EAAT2 (193).

EAAT4 is predominantly found in the cerebellar Purkinje cells (66, 123) where it is targeted to the dendrites, the spines in particular (123), but there is also some EAAT4 in a subset of forebrain neurons (123, 207, 208).

Outside the CNS, EAAT2 is primarily expressed in glandular tissue, including mammary gland, lacrimal gland, and ducts and acini in salivary glands (212) and by perivenous hepatocytes (212, 213). It is not present in the heart (214). Thus, the main roles of EAAT2 are in the brain [for review, see Ref. (3, 15, 16)]. EAAT1 is found in several non-neuronal tissues (212) including, the heart, fat cells, and taste buds (212, 214, 215), but does not appear to be important in controlling bone growth (216). EAAT3 is present in the kidney (62, 193). The heart expresses EAAT1, EAAT3, EAAT4, and EAAT5, but not EAAT2 (214).

### Neuronal expression of the EAAT2 protein

EAAT2 mRNA is detected in astrocytes, but is also found in some neurons: pyramidal cells in CA3 hippocampus and in layer VI of the parietal neocortex (217–219). In fact, EAAT2 mRNA is reported in the majority of neurons in the neocortex and in parts of the olfactory bulb, thalamus, and inferior olive (188). That neurons have the potential to express EAAT2 protein is clear. Cultured neurons from hippocampus and cortex can express EAAT2 protein (220–222). Further, EAAT2 is transiently localized on growing axons of the mouse spinal cord before establishing astrocytic expression (223). Finally, in the normal and mature mammalian retina, EAAT2 protein is not expressed in retinal glial cells (neither in the Müller cells nor the astrocytes), but is exclusively expressed in neurons (cone photoreceptors and bipolar cells) (201, 205, 224–226).

Nevertheless, this was controversial for a long time and remains to be fully resolved. What is clear is that there is a significant glutamate uptake into glutamatergic nerve terminals, at least in the hippocampus CA1 [Ref. (227) for a review, see section 4.2 in Ref. (3)]. Glutamatergic axon terminals in hippocampus CA1 express EAAT2 protein, albeit at low levels (154, 228–231) and this transporter is responsible for the glutamate uptake activity in hippocampal terminals because it is absent in EAAT2 knockout mice and is sensitive to inhibition by dihydrokainate (193, 229). Because these terminals originate from the CA3 pyramidal cells, it makes sense that these cells have high levels of EAAT2 mRNA. This is further confirmed by the recent observation from EAAT2 eGFP BAC reporter mice (232). There is now consensus up to here, but at least two questions remain:

(a) Is nerve terminal glutamate uptake functionally relevant? Why was about half of all d-aspartate taken up by hippocampus slices found to in axon terminals when terminals only contain around 10% of the EAAT2 protein (229)? This uptake cannot simply be disregarded as an *in vitro* artifact due to a higher rate of heteroexchange than net uptake (233). Preliminary data from selective deletion of EAAT2 in axon terminals indicate disturbances in synaptic transmission (234), and thereby may suggest that EAAT2 in terminals is functionally relevant.
(b) Do CA3 pyramidal neurons represent special cases or is most of the so called synaptosomal uptake measured in other brain regions also due to nerve terminal EAAT2?

Data obtained with EAAT2 eGFP BAC reporter mice (232) tend to favor a “yes” to this question, while *in situ* hybridization data argue for a “no” [e.g., Ref. (154, 235)].

## Lessons from GABA Transporter Knockouts

GAT1-deficient mice were generated as an intermediate in the construction of the mGAT1-GFP strain (236). As GAT1 is the major GABA transporter, one might expect that deletion would lead to increased extracellular GABA levels and inhibition. Reduced aggression (237), hypoalgesia (238), reduced anxiety, and depression-like behaviors (239) and altered behavioral responses to ethanol (240) may be largely as expected. However, things are more complicated. One complicating factor is that the brain still expresses GAT3 in astrocytes. Another point is that GAT1 is mostly in terminals where it recycles GABA, and GAT1 deletion leads to decreased quantal GABA release, and a differential tonic activation of GABA(A) versus GABA(B) receptors in the hippocampus (241), as well as to tremor, ataxia, nervousness, and increased GABA-induced tonic conductance in cerebellum (242). This phenotype resembles adverse effects of tiagabine treatment. Tiagabine is highly selective for GAT1 (115). It has effects on seizure control and behavior, but side effects are fatigue, dizziness, psychomotor slowing, ataxia, gastrointestinal upset, weight change, and insomnia (243). In human populations there is genetic variation within the GAT1 gene (slc6a1) and these may be associated with anxiety disorders with panic symptoms (244).

Deletion of BGT1 in mice does not affect seizure thresholds (corneal kindling; minimal clonic and tonic extension threshold test; 6 Hz seizure threshold test; pentamethylenetetrazole-induced seizure) of adult mice (167) in agreement with the fact that it is predominantly expressed in the liver and at lower levels in the kidneys and at the brain surface (106). Deletion of GAT2 in mice leads to changes in liver and brain taurine contents (181), but also does not appear to give any symptoms from the nervous system under non-challenging rearing conditions (181). Obviously, it would be interesting to study the consequences of the deletion of GAT3. These studies are under way as GAT3 knockout mice have been made by Yun Zhou, C. Guo, and Niels Christian Danbolt.

## Lessons from Glutamate Transporter Knockouts

The possibility of connections between malfunctioning glutamate transporters and disease has got considerable attention [e.g., for review, see Ref. (3, 74, 245, 246)]. Observations of the EAAT2 knockout mice illustrate why (190, 191). Deletion of the EAAT2 gene causes, in agreement with biochemical data (59, 129), a reduction in glutamate uptake activity by about 95% (155, 190, 192, 193) and increased extracellular glutamate levels (247, 248). This has dramatic consequences as they grow up. Mice deficient in EAAT2 are not conspicuous at birth, but at 3 weeks of age they can readily be identified because they are hyperactive, epileptic, and smaller than their wildtype littermates. About half of the mice die from spontaneous seizures before they reach 4 weeks of age (190). The gradual increase in severity parallels the postnatal increase in EAAT2 expression in wildtype animals (249, 250), and in production of transmitter glutamate via the glutamate-glutamine cycle [reviewed by Ref. (251)]. The heterozygote EAAT2 knockout mice (±) exhibit a 59% decrease in EAAT2 protein levels in the brain, but do not show any apparent morphological brain abnormalities and have a similar life-span as their wildtype littermates (192). There are only moderate behavioral alterations (mild sensorimotor impairment, hyperlocomotion lower anxiety, better learning of cue-based fear conditioning), but worse context-based fear conditioning (192). However, the histological outcomes following traumatic spinal cord injury is worse in agreement with the notion that they are less protected (252). No humans have been identified at being EAAT2 deficient, but there are some reports on mutations. One patient with amyotrophic lateral sclerosis was found to harbor a mutated EAAT2 (253, 254) and associations of mutations with alcoholism (255), schizophrenia (256), smoking behavior (257), essential tremor (258), and bipolar disorder (259) have been reported, but it is too early to make firm conclusions.

Mice lacking EAAT1 (260) develop normally, but show symptoms of insufficient glutamate uptake in regions where EAAT1 is the major glutamate transporter. Thus, cerebellar function is affected resulting in reduced motor coordination and increased susceptibility to cerebellar injury (260), disturbance of the inner ear with exacerbation of noise-induced hearing loss (261) and disturbed retinal function (262). The EAAT1 knockout mice also display poor nesting behavior; abnormal sociability, reduced alcohol intake, and reward (260, 263–265). Lack of GLAST does not lead to spontaneous seizures like those seen in connection with EAAT2-deficiency, but when seizures are initiated, then lack of GLAST increases seizure duration and severity (266). In humans, mutations in EAAT1 are associated with episodic ataxia (246, 267, 268).

Mice lacking EAAT3 (269) develop dicarboxylic aminoaciduria, and possibly a somewhat reduced spontaneous locomotor activity (open field). They do not show signs of neurodegeneration at young age and do not have epilepsy (269–271), but may age prematurely (270). Humans lacking EAAT3 develop dicarboxylic aminoaciduria as expected from the mice data (272). Further, human EAAT3 polymorphisms have been reported to be associated with obsessive–compulsive disorders (273, 274).

## Concluding Remarks

The various GABA and glutamate transporters have select expression patterns and distributions. The literature, however, has become confusing in part due to poorly controlled immunocytochemistry. A major reason for the latter is the reliance on the pre-absorption test which easily gives a misleading impression of specificity [for discussion, see Ref. (149)]. Post-mortem proteolysis has also contributed to confusion concerning distributions in humans (152). To sum up (for references, see above): GAT3 and EAAT1 (GLAST) are both selectively expressed in astrocytes throughout the CNS, while EAAT3 (EAAC1) and EAAT4 are selective for neurons. EAAT3 is expressed by most if not all neurons, while EAAT4 is only expressed in subpopulations. GAT1 and EAAT2 (GLT-1) are both in terminals (GABAergic and glutamatergic, respectively) and in astrocytes, but differ in that EAAT2 is predominantly in astrocytes throughout the CNS except in the retina, while GAT1 is only predominantly astrocytic at some locations (e.g., thalamus). EAAT2 is the only one of these transporters that is required for survival under non-challenging conditions. GAT2 and BGT1 are both expressed in the leptomeninges, but are not significantly expressed not around synapses (in neuropil). GAT2 is also found in some blood vessels. All these transporters are highly conserved between mammals, and they play different roles, some of which remains to be fully understood.

## Conflict of Interest Statement

The authors declare that the research was conducted in the absence of any commercial or financial relationships that could be construed as a potential conflict of interest.

## Abbreviations

BGT1, betaine-GABA transporter (slc6a12); DTT, dithiothreitol; EAAC1, excitatory amino acid carrier (EAAT3; slc1a1); EAAT, excitatory amino acid transporter; GAT1, GABA transporter 1 (slc6a1); GAT2, GABA transporter 2 (slc6a13); GAT3, GABA transporter 3 (slc6a11); GABA, gamma-aminobutyric acid; GLAST, glutamate–aspartate transporter (EAAT1; slc1a3); GLT-1, glutamate transporter 2 (EAAT2; slc1a2).
